# Exploring qualitative methods reported in registered trials and their yields (EQUITY): systematic review

**DOI:** 10.1186/s13063-018-2983-y

**Published:** 2018-10-29

**Authors:** Clare Clement, Suzanne L. Edwards, Frances Rapport, Ian T. Russell, Hayley A. Hutchings

**Affiliations:** 1 Bristol Medical School, University of Bristol, Canynge Hall, 39 Whatley Road, Bristol, UK; 2 Swansea University Medical School, Swansea University, Swansea, UK; 3 Australian Institute of Health Innovation, Centre for Healthcare Resilience and Implementation Science, Macquarie University, Sydney, Australia

**Keywords:** Qualitative research, Randomised controlled trial, Research design, Registries, Income, Humans, Research support

## Abstract

**Background:**

The value of qualitative methods within trials is widely recognised, but their full potential is not being realised. There are also issues with the visibility, recognition and reporting of qualitative methods in trials. To identify potential improvements in qualitative research within trials, we need to study trials that have included qualitative methods. We aimed to explore the frequency of reporting qualitative methods in registered trials, the types of trials using qualitative methods and where in the world these trials were conducted.

**Methods:**

We included registries if they were searchable using keywords and held summaries of trials rather than listing reports or publications. We searched the included registries from the first available record in 1999 to the end of 2016 for the term ‘qualitative’. We included trials only if we could confirm that they used qualitative methods through documented use of qualitative data collection and analysis in the registry summary. We analysed registered trials reporting the use of qualitative methods by: year registered, the country responsible for overseeing governance of the trial and the type of trial intervention (categorised as surgical, medical device, behavioural, drug or other).

**Results:**

We included three registries: ClinicalTrials.gov, the International Standard Randomised Controlled Trial Number Registry (ISRCTN) and the World Health Organisation International Clinical Trials Registry Platform (WHO ICTRP). A total of 615,311 trials appear in these three registries from 1999 until the end of 2016. Numbers differed across registries with the WHO ICTRP the largest (366,753 trials), ClinicalTrials.gov the second largest (233,277) and ISRCTN the smallest (15,301). Of these registered trials, we confirmed that 1492 (0.24%) reported using qualitative methods. The ISRCTN contributed the highest percentage of trials reported as using qualitative methods (3.4%); in contrast, ClinicalTrials.gov reported 0.3% and WHO ICTRP reported 0.03%. The number and percentage of trials reported to use qualitative methods increased over time from 0 (0.0%) in 1999 to 285 (0.38%) in 2016. Trials reported as using qualitative methods originated from 52 countries across the world. Most were in Western higher-income countries: 38% in the United Kingdom and 28% in the United States. Most registered trials reported as using qualitative methods evaluated behavioural (39%) or other interventions with many fewer trials evaluating drugs (5%), medical devices (5%) or surgical interventions (4%).

**Conclusion:**

The reported use of qualitative methods in registered trials has increased over time and worldwide. They are reportedly more frequent in high-income countries and in trials of behavioural and other interventions. Trialists and other stakeholders need to recognise the benefits of using qualitative methods in surgical, device and drug trials, and trials conducted in poorer countries. Moreover, they should seriously consider using qualitative methods in these trials.

## Background

The added value of using qualitative research methods within trials is widely recognised [1–6]. O’Cathain et al. [1] identified 22 ways in which qualitative research could benefit trials including: identifying and addressing recruitment and retention issues [4], ensuring trial designs are appropriate to the population and condition they are addressing [7] and facilitating the interpretation and implementation of trial findings through understanding trial context [8]. Qualitative methods can also assess whether trial processes are appropriate [9]. These methods have informed trial design and conduct, for example, by assessing fidelity and uptake of interventions and how and why they work or not [10, 11]. Randomised trials are the most appropriate design for robust evaluations of complex health interventions [12, 13]. However, they are not without criticism. There are concerns that they neglect patient and professional input [14] and are insensitive to the health-care context [15]. Critics see them as artificial constructs that depart from the real world [16] and therefore, cannot model health-care on the ground [17].

Hence, trialists have turned to complementary methods to address these concerns. Methodological guidance [18, 19] and mounting evidence of the added value conferred by qualitative methods in trials [1, 3, 20, 21] have led trialists increasingly to adopt a multi-method approach, integrating quantitative and qualitative components. While qualitative methods are increasingly used within trials, their full potential is often not realised [22]. They have not always been well integrated into trial designs, which reduces methodological rigour and transparency [3]. For example, excluding qualitative methodologists from the design phase, especially from the formulation of trial aims and objectives, may create conflict between qualitative and quantitative components. Poor integration at the design stage often leads to poor reporting that obscures qualitative methods and findings [22, 23]. To identify how to improve qualitative methods within trials, it is important to analyse how trials report the use of qualitative methods and whether these have changed over time.

Previous reviews have reported when, where and how qualitative methods are used within trials [3, 20]. Though the reported numbers of trials employing qualitative methods differ across reviews, they are consistently low compared with the total number conducted and published. The proportion of trials that have reported qualitative methods varies from 1% in palliative care trials [20] to 30% in trials of complex interventions [3]. The reviews also reveal that, though trials including qualitative methods are conducted worldwide with multi-national authorships, they are mainly within rich countries [3, 22]. However, these reviews are essentially cross-sectional, encompassing a couple of years, for example 2008–2010 [1]. Moreover, searches cover only single registries or published trials. Furthermore, these reviews have focused mainly on trials of complex interventions that evaluated behavioural interventions aimed at changing participants’ behaviour at the individual or community level [20, 24]. More recently the term ‘complex intervention’ has evolved to cover a wider range of interventions, including surgical procedures, medical devices and drugs. This reflects the increasing complexity of clinical interventions [7, 19, 25]. It is, therefore, important to subdivide these complex interventions from previous reviews to characterise the trials that report qualitative methods.

Depending on the country of the sponsor, clinical trials are either required or encouraged to register prospectively with a trials registry. These registries have been established across the world to address concerns about access to trials, publication bias and more recently, trial results. In the United States, the Food and Drug Administration Modernization Act of 1997 and subsequent amendments mandated the development of a registry and registration of both federally funded and privately funded trials, with penalties for non-compliance [26]. In 2004, the members of the International Committee of Medical Journal Editors published an editorial promoting the prospective registration of all clinical trials, leading to the establishment of a trial registry within the United Kingdom [27]. This was later supported by the World Health Organisation (WHO), which promoted registration further afield [28]. Registries aim to provide increased access to information and transparency about trials for researchers, clinicians, patients and members of the public. These registries give access to information about each trial provided by the trial team, including: lead researcher’s name and organisation, study design including type of trial and methods, and the organisation responsible for overseeing governance. In principle, they also report the extent of qualitative methods within the trial.

This review is part of a larger project to characterise best practice in conducting qualitative research within trials. The objectives of this project are:

1. To describe the characteristics of trials reporting the use of qualitative methods
2. To explore good practice in planning and running clinical trials using qualitative methods
3. To explore the roles that participants play in clinical trials using qualitative methods
4. To explore and identify potential facilitators of and barriers to qualitative research within trials
5. To make recommendations for best practice for using qualitative methods within trials.

This review builds on previous reviews by estimating the frequency of the reported use of qualitative methods in trials over 16 years, longer than previous reviews, and analysing trials that report using qualitative methods, specifically the types of intervention evaluated and their locations.

## Methods

To assess the use of qualitative methods in trials, we reviewed existing clinical trial registries and identified trials that reported using qualitative methods, in four main steps:

Step 1: We used internet search engines to identify existing clinical trial registries. We included registries if: they could be searched for keywords; they held records of individual trials, not merely reports or publications; and they held records in English. We searched all included registries from the first available record, which varied across registries, until 31 December 2016.

Step 2: We searched these registries for the keyword ‘qualitative’. The lead researcher (CC) reviewed all identified trials and extracted the following data into an Excel spreadsheet: registry name (to allow comparison across registries), registry record number (as a unique identifier), trial title, year of first registration with registry, country responsible for overseeing governance of the trial (as many trials recorded multiple recruiting countries, we chose the most likely source of decisions about trial design) and type of trial intervention (categorised as surgical, medical device, drug, behavioural – which aimed to modify the behaviour of individuals or communities – or other). We derived these types from descriptions used by the registries, existing literature and previous reports [22, 29, 30].

Step 3: We checked the registry records for documented use of qualitative methods. We defined these as qualitative data collection (such as observation, interviews, focus groups, documents or visual data), qualitative data analysis (such as textual or visual) or both [31, 32].

Step 4: We analysed these data using the filter and count features within Excel. We counted frequencies for: number of registered trials reporting the use of qualitative methods, year of first registration with registry, country responsible for overseeing governance of the trial and type of trial intervention as defined in Step 2. We presented our findings as frequencies and percentages.

## Results

### Trial registries

Our search identified five main clinical trial registries: ClinicalTrials.gov, WHO’s International Clinical Trial Registry Platform (WHO ICTRP), the International Standard Randomised Controlled Trial Number (ISRCTN) Registry, the Cochrane Central Register of Controlled Trials (Cochrane CENTRAL) and the European Union Clinical Trials Register. However, we excluded the last of these, as it forms part of the WHO ICTRP, and Cochrane, as it is a database of trial reports rather than a registry of trials.

### Included registries

#### ClinicalTrials.gov

This registry was created in response to patient pressure for access to information on clinical trials. It is run by the United States National Library of Medicine within the National Institutes of Health and claims to be the largest clinical trials database in the world, registering trials from 200 countries [30]. It records information on federally, commercially and privately funded clinical trials, including information on participant eligibility, locations of trial activity, point of contact and, more recently, basic results. US law enforces penalties for non-compliance with this registry. Approximately 38% of the trials registered within ClinicalTrials.gov are based only inside the US, 56% are based only outside the US and 5% are based in both [30].

#### International Standard Randomised Controlled Trial Number Registry

The ISRCTN registry contains basic data on all clinical trials which have been assigned an ISRCTN number. The registry is a not-for-profit organisation sponsored by the Canadian Institute of Health Research, the Italian Instituto di Ricerche Farnacologiche ‘Mario Negri’, the Netherlands Organisation for Health Research and Development, the UK Department of Health, and the UK Medical Research Council. However, most of the registered trials are based in the UK [33]. The ISRCTN is a simple numeric system that facilitates the identification and tracking of trials throughout their life cycle. The registry uses the WHO 20-item Trial Registration Data Set covering: study hypothesis, study design, countries of recruitment, selection criteria, disease or condition, intervention, sponsor and contact information [33].

#### World Health Organisation International Clinical Trials Registry Platform

This registry also uses the WHO Trial Registration Data Set. The portal provides access to 16 separate registries from across the world [34], including ClinicalTrials.gov and ISRCTN. Thus, we took care not to duplicate trials from those registries.

#### Trials with confirmed use of qualitative methods

The three included registries recorded a total of 615,311 trials from their first record (occurring in 1999 for ClinicalTrials.gov, 2004 for ISRCTN and 2006 for WHO ICTRP) until 31 December 2016. The WHO ICTRP registry was the largest with 366,753 trials registered, ClinicalTrials.gov the second largest with 233,277 trials and ISRCTN the smallest with 15,301 trials. Of these, 2477 records included the keyword ‘qualitative’: 144 (0.03%) from WHO ICTRP, 1668 (0.7%) from ClinicalTrials.gov and 665 (4.6%) from ISRCTN. Of these 2477 records, we confirmed that 1492 (60.2%) trials had used qualitative methods. The main reasons for excluding 985 records were: use of the term ‘qualitative’ to describe quality of life measures, to refer to medical tests like ‘qualitative urine test or MRI imaging’ or to cite statistical tests as ‘qualitative Fishers Exact Test’. None of these fitted our criteria for qualitative methods.

Table 1 shows that ISRCTN contributed by far the highest percentage of registered trials subsequently confirmed as using qualitative methods (3.4%). In contrast, ClinicalTrials.gov had only 0.3%, and WHO ICTRP had the smallest proportion at 0.03%.

**Table 1:** Registered trials using qualitative methods by registry

	WHO ICTRP	ClincalTrials.gov	ISRCTN	Overall
Total trials in registry from 1999 to 2016	366,753	233,277	15,301	615,311
Total identified with qualitative keyword	144	1668	665	2477
Total records excluded	46	790	149	985
Total confirmed with qualitative methods	98	878	516	1492
Percentage confirmed with qualitative methods	0.04%	0.4%	3.4%	0.2%

*ICTRP* International Clinical Trials Registry Platform, *ISRCTN* International Standard Randomised Controlled Trial Number Registry, *WHO* World Health Organisation

#### Trials confirmed as using qualitative methods by year registered

**Fig. 1:**
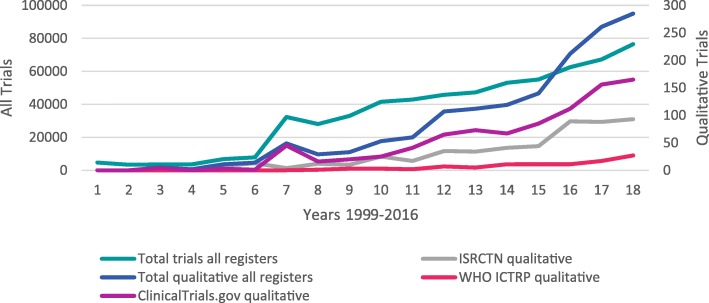
Number of registered trials confirmed as using qualitative methods by registry by year. ICTRP International Clinical Trials Registry Platform, ISRCTN International Standard Randomised Controlled Trial Number Registry, WHO World Health Organisation

**Fig. 2:**
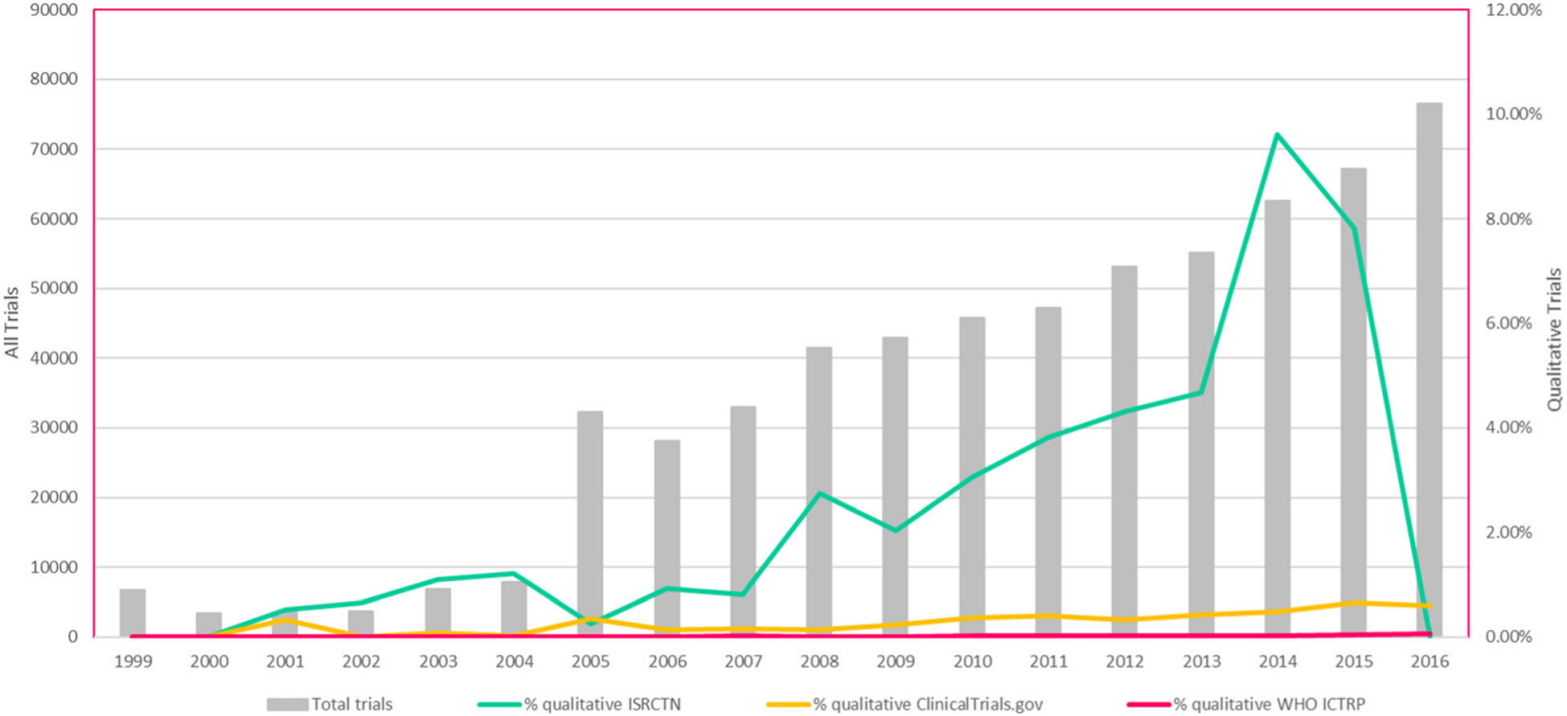
Registered trials confirmed as using qualitative methods by registry by year. ICTRP International Clinical Trials Registry Platform, ISRCTN International Standard Randomised Controlled Trial Number Registry, WHO World Health Organisation

The number of registered trials increased over time from 1999, when first reported in ClinicalTrials.gov, to the end of 2016. The number and percentage of these trials reported as having used qualitative methods also increased steadily over time across all registries (Figs. 1 and 2). The year in which the first trial reported to use qualitative methods was identified differed across the registries: 2000 in ISRCTN, 2001 in ClinicalTrials.gov and 2006 in WHO ICTRP. As all registries held records of trials reported as using qualitative methods from 2004, we compared the number across time within each registry between 2004 and 2016. This revealed substantial increases across time in all three registries: from 1.2% to 8.4% in ISRCTN, from 0.03% to 0.59% in ClinicalTrials.gov and from 0% to 0.06% in WHO ICTRP.

#### Types of registered trials confirmed as using qualitative methods

Of the 1492 registered trials confirmed as reporting the use of qualitative methods, most were evaluating a behavioural intervention (39%) or an other intervention that did not fit the defined categories, mainly vaccines, nutritional supplements and diagnostic testing (47%). In contrast, clinically orientated trials evaluating drugs (5%), medical devices (5%) or surgical interventions (4%) were much less likely to report the use of qualitative methods. This was broadly consistent across the three trial registries (Table 2).

**Table 2:** Registered trials confirmed as using qualitative methods by type of intervention by registry

Type of trial intervention	WHO ICTRP	ClinicaTrial.gov	ISRCTN	Total
Other	75 (76.5%)	335 (38.2%)	289 (56.0%)	699 (46.9%)
Behavioural	20 (20.4%)	419 (47.7%)	147 (28.5%)	586 (39.3%)
Drug	2 (2%)	43 (4.9%)	37 (7.2%)	82 (5.5%)
Medical device	1 (1%)	54 (6.2%)	14 (2.7%)	69 (4.6%)
Surgical	0 (0%)	27 (3.1%)	29 (5.6%)	56 (3.8%)

*ICTRP* International Clinical Trials Registry Platform, *ISRCTN* International Standard Randomised Controlled Trial Number Registry, *WHO* World Health Organisation

#### Registered trials confirmed as using qualitative methods by country

Trials with confirmed use of qualitative methods were registered from 52 countries across the world. The highest number were registered in the UK (570 trials, 38.2%), followed by the US (425 trials, 28.5%), Canada (71 trials, 4.6%), France (67 trials, 4.5%), Australia (43 trials, 2.9%), Germany (37 trials, 2.5%) and Denmark (34 trials, 2.3%). None of the remaining 45 countries accounted for more than 2% of all confirmed qualitative trials.

We examined each registry for the country overseeing most of the registered trials reported to use qualitative methods. Most of the trials registered within ISRCTN were conducted in the UK (444, 77.9%); 124 UK trials (21.8%) were registered in ClinicalTrials.gov and two UK trials in WHO ICTRP. Most of the trials within ClinicalTrials.gov were conducted in the US (419, 98.6%), with six US trials (1.4%) in ISRCTN but no US trials (0%) in WHO ICTRP. Most of the trials within WHO ICTRP were conducted in Australia (36, 15.5%), with four Australian trials (9.3%) in ClinicalTrials.gov and three Australian trials (6.8%) in ISRCTN.

We classified countries by gross national income (GNI), which was formerly known as gross domestic product (GDP), as estimated by the World Bank Group using the World Bank Atlas Method [35]. Most registered trials reported to use qualitative methods were conducted in high-income countries like the UK (570 trials, 38.2%) and the US (425 trials, 28.5%). Low- and low-middle-income countries had very few trials reported as using qualitative methods, for example Uganda (four trials, 0.26%) and Ethiopia (two trials, 0.13%) (Table 3). In all registries, most of the trials that reported using qualitative methods were in the high-income category. However, the distributions for each category differ across registries: most of the trials that reported using qualitative methods within low-income or low-middle-income countries were registered with ClinicalTrials.gov.

**Table 3:** Registered trials confirmed as using qualitative methods by country income by registry

Country income category	Gross national income	WHO ICTRP	ClinicalTrials.gov	ISRCTN	Total
Low	$1005 or less	0 (0%)	14 (73.6%)	5 (26.3%)	19 (1.3%)
Low-middle	$1006–$3955	2 (7%)	21 (72.4%)	6 (20.7%)	29 (1.9%)
Upper-middle	$3956–$12,235	5 (11%)	41 (89.1%)	1 (2.17%)	47 (3.1%)
High	$12,236 or more	91 (7%)	802 (57.4%)	504 (36.1%)	1397 (93.7%)

*ICTRP* International Clinical Trials Registry Platform, *ISRCTN* International Standard Randomised Controlled Trial Number Registry, *WHO* World Health Organisation

## Discussion

### Summary

This review has characterised trials registered on trial registries and confirmed as using qualitative methods, both across time (between 1999 and 2016) and across countries. Only 1492 (0.24%) of the 615,311 registered trials identified across the three included trial registries, either completed or in progress, reported the use of qualitative methods. Most of these were based in the US or UK, rich Western counties where the number of trials reported to use qualitative methods has increased steadily over time. Most trials reporting the use of qualitative methods investigated behavioural or other interventions, while trials evaluating drugs, medical device or surgical procedures each contributed fewer than 5% of registered trials reported to use qualitative methods.

### Interpretation

Our finding that reported use of qualitative methods is rare amongst clinical registered trials is consistent with O’Cathain et al. [22], who found that few published drug or medical device trials employed qualitative methods. Surgical trials are reputedly difficult to design and conduct, so until recently, surgeons resisted the use of randomised trials [25]. Although the number of surgical trials being conducted is increasing [25, 36], they face challenges; in particular the beliefs and preferences of participating surgeons threaten their equipoise, that is whether they are genuinely uncertain about the effectiveness of a clinical intervention [37]. Many surgeons prefer not to standardise interventions, which contributes to good trial design [38]. However, qualitative methods can describe experiences and beliefs and help in the understanding of complex phenomena. They can explore factors affecting equipoise and how to overcome these, and help to establish core outcomes and minimum standards for interventions [7]. Hence, qualitative methods can describe surgical behaviour and explore recruitment issues in surgical trials. For example, Donovan and colleagues developed the Qualitative Recruitment Intervention [4], which has been implemented in surgical trials [39]. However, qualitative methods remain rare in clinical trials and research is needed to explore why.

As drug trials are better established, it is unclear why they too rarely report qualitative methods. There is evidence of the benefits of qualitative methods in drug trials, for example in understanding, identifying and addressing barriers to recruitment [4], and exploring equipoise [8]. As medical devices are increasing in variety and complexity [40], there is a strong case for evaluating their benefits and harms through trials [41]. Such trials face similar issues to surgical trials, including: when to initiate trials, when to assess outcomes, the acceptability of the intervention, the choice of outcome measures and how to implement devices into routine practice [41]. Qualitative methods can help to tease out these issues, especially by conceptualising core outcomes [42], showing how medical devices are perceived and integrated into existing practice [40], and exploring the most appropriate trial design [1]. For example, a qualitative consultation with key stakeholders, notably patients and professionals, can illuminate decisions about: trial arms, study outcomes (in particular, clinical versus patient reported) and frequency of reporting.

It is important to consider why registered trials of behavioural interventions are more likely to report using qualitative methods, as this could help to increase their use in more clinical trials. One plausible explanation is that qualitative research methods emerged from the behaviourally oriented social sciences and humanities. Such disciplines have generally avoided positivism, the dominant epistemology in biomedical sciences, in favour of interpretivism and constructionism to understand how and why people behave the way they do [43]. Hence, using qualitative methods may be more acceptable in trials that evaluate behavioural interventions. So, advancing the use of qualitative methods in other trials may depend on convincing their researchers of the benefits of the interpretivist approach. However, this hypothesis needs careful investigation.

The continuing increase in reported use of qualitative methods in registered trials may indicate increased awareness of qualitative methods or of the potential benefits of including them in trials. Publications that may have contributed to this increase include the empirical work of O’Cathain [1], Lewin [3] and Flemming [1, 3, 20] and guidance on using qualitative methods in trials [1, 2, 22, 44, 45]. As these publications primarily addressed British trials, this may account for the greater use of qualitative methods in the UK and the UK-based ISRCTN registry.

Pragmatic randomised trials seek to evaluate interventions in normal clinical practice and thus, allow more clinical autonomy. This yields more insight into participants’ views and experiences during the trial and a more representative picture of lived experiences and real practice [46]. Thus, more pragmatic trials are using qualitative methods to characterise patient experiences and clinical practice beyond the trial [1, 47]. Increased adoption of pragmatic trials may also have increased the use of qualitative methods.

This review has found that, though relatively few registered trials report using qualitative methods worldwide, most of these are conducted within rich Western countries, consistent with previous reports [3, 22]. There have been calls for more trials within poorer countries, which have a greater potential to improve public health [48]. However, obstacles to such trials include: less capacity to deliver trials, weaker links between trial conduct and current practice, and the need to adapt trials to local context and culture. These issues make qualitative methods even more challenging [48, 49]. Nevertheless, Vischer and colleagues [48] have shown how qualitative methods can address these issues in low-income countries. They interviewed key informants in Burkina Faso, Ghana, Kenya and Senegal to investigate factors slowing clinical trials. Trial staff described factors apparently hindering trials, including lack of planning and poor understanding of trial processes. This generated recommendations for explicit trial planning and site organisation [48]. Thus, qualitative methods can improve the conduct of trials in poorer countries, such as by consulting stakeholders, not least about cultural acceptability, for example of trial outcome measures. It is important, therefore, to test whether applying this approach more widely can both increase the number of trials and the proportion that use qualitative methods. It is also important to disseminate such work through publication in international journals and rigorous training.

### Strengths, limitations and future directions

This review is limited to trials reported by researchers in trial registries as using qualitative methods and confirmed by inspecting their registry summaries. However, there may be registered trials that use qualitative methods without reporting this to the registry. Indeed, searching the three registries for trials using the terms ‘interviews’, ‘focus groups’ or ‘mixed methods’ identified 8267 registered trials. We checked a random 177 of these and found that 50 of their registry summaries reported the use of qualitative methods. Hence, the true number of clinical trials in these registries using qualitative methods is closer to 3800 (0.62%). This highlights two issues: Why did registries not check trials a little more thoroughly for use of qualitative components? How should registries identify trials with qualitative methods in future? We recommend that, as more trials use qualitative methods, trial registries should ask about qualitative methods within their application forms.

We did not address whether registries reported findings or whether qualitative methods influenced trial processes, outcomes or plans for implementation. With increasing pressure on trials to report findings within registries, now mandated within the US, it will soon be possible to see whether trials report qualitative findings and, in particular, whether they use qualitative methods to interpret findings or alter trial design. It will also become important to examine how registries apply those methods. Although they collect similar data, they differ in how they register trials and manage data, and above all in the proportion of trials that report the use of qualitative methods.

This review reports on important characteristics of registered trials that reported using qualitative methods, namely: when they registered, where they were conducted and the type of intervention they evaluated. Unfortunately, information was limited and inconsistent about other trial features, notably the design of the registered trials, their use of qualitative methods, their phase, sample sizes for both trials and their qualitative studies, trial outcome measures (for example, the balance between clinical and patient-reported), qualitative methods used, and how these methods related to trial objectives. While much of this information is available in the corresponding peer-reviewed publications, extracting it is a major task, as is analysing the relationship between these characteristics and trials’ use of qualitative methods.

A strength of this review is the inclusion of all trials registered between the start of 1999 and the end of 2016. This has shown a clearly increasing trajectory of trials using qualitative methods. Previous reviews covered shorter periods of time and could not analyse changes over time [1, 3]. Including the three main international registries has improved our understanding of when and where trials are using qualitative methods.

## Conclusion

This review has highlighted the increasingly reported use of qualitative methods in registered trials over time and across countries. However, these methods are more prevalent in rich Western countries and in less clinically orientated trials. Trialists and other stakeholders need to recognise the benefits of using qualitative methods in surgical, device and drug trials, and trials conducted in poorer countries, and should seriously consider the use of qualitative methods in these trials.
